# Validity, reliability, and measurement invariance of an adapted short version of the HIV stigma scale among perinatally HIV infected adolescents at the Kenyan coast

**DOI:** 10.1186/s41256-021-00229-9

**Published:** 2021-12-10

**Authors:** Stanley W. Wanjala, Derrick Ssewanyana, Patrick N. Mwangala, Carophine Nasambu, Esther Chongwo, Stanley Luchters, Charles R. J. C. Newton, Amina Abubakar

**Affiliations:** 1 Department of Public Health and Primary Care, Ghent University, Ghent, Belgium; 2 Department of Social Sciences, Pwani University, Box 195, Kilifi, Kenya; 3 Neuroassessment Group, KEMRI/Wellcome Trust Research Programme, Centre for Geographic Medicine Research (Coast), Kilifi, Kenya; 4 Alliance for Human Development, Lunenfeld-Tanenbaum Research Institute, Toronto, Canada; 5 Institute for Human Development, Aga Khan University, Nairobi, Kenya; 6 Department of Epidemiology and Preventive Medicine, Monash University, Melbourne, Australia; 7 Department of Population Health, Medical College, Aga Khan University, Nairobi, Kenya; 8 Department of Public Health, Pwani University, Kilifi, Kenya; 9 Department of Psychiatry, University of Oxford, Oxford, UK

**Keywords:** Stigma, Adolescents, HIV/AIDS, Psychometrics, Measurement invariance, Kenya

## Abstract

**Background:**

There is a dearth of instruments that have been developed and validated for use with children living with HIV under the age of 17 years in the Kenyan context. We examined the psychometric properties and measurement invariance of a short version of the Berger HIV stigma scale administered to perinatally HIV-infected adolescents in a rural setting on the Kenyan coast.

**Methods:**

A cross-sectional study was conducted among 201 perinatally HIV-infected adolescents aged 12–17 years between November 2017 and October 2018. A short version of the Berger HIV stigma scale (HSS-40) containing twelve items (HSS-12) covering the four dimensions of stigma was evaluated. The psychometric assessment included exploratory factor analysis, confirmatory factor analysis (CFA), and multi-group CFA. Additionally, scale reliability was evaluated as internal consistency by calculating Cronbach’s alpha.

**Results:**

Evaluation of the reliability and construct validity of the HSS-12 indicated insufficient reliability on three of the four subscales. Consequently, Exploratory Factor Analysis (EFA) was conducted to identify problematic items and determine ways to enhance the scale’s reliability. Based on the EFA results, two items were dropped. The Swahili version of this new 10-item HIV stigma scale (HSS-10) demonstrated excellent internal consistency with a Cronbach alpha of 0.86 (95% confidence interval (CI) 0.84–0.89). Confirmatory Factor Analysis indicated that a unidimensional model best fitted the data. The HSS-10 presented a good fit (overall Comparative Fit Index = 0.976, Tucker Lewis Index = 0.969, Root Mean Square Error of Approximation = 0.040, Standardised Root Mean Residual = 0.045). Additionally, multi-group CFA indicated measurement invariance across gender and age groups at the strict invariance level as ΔCFI was ≤ 0.01.

**Conclusion:**

Our findings indicate that the HSS-10 has good psychometric properties and is appropriate for evaluating HIV stigma among perinatally HIV-infected adolescents on the Kenyan coast. Further, study results support the unidimensional model and measurement invariance across gender and age groups of the HSS-10 measure.

## Background

Globally, an estimated 1.7 million adolescents (10–19 years) were living with the Human Immunodeficiency Virus (HIV) in 2019, and almost 88% of them live in sub-Saharan Africa [1]. The improved access to antiretroviral therapy (ART), especially in resource-constrained settings, has significantly boosted perinatally HIV-infected children's survival. Subsequently, many of these children have transitioned into adolescence and older age groups [2, 3], although HIV-related challenges, such as stigma, continue to negatively impact their well-being [4].

Erving Goffman [5] defines stigma as an *attribute that is deeply discrediting" and reduces a person "from a whole and usual person to a tainted discounted one*. Individuals living with HIV experience stigma through three inter-related mechanisms: anticipated stigma, internalised stigma, and enacted stigma [6]. Anticipated stigma refers to the extent to which individuals living with HIV expect to experience discrimination and prejudice from other people in the future [7]. Internalised stigma refers to the extent to which individuals living with HIV approve of the negative feelings and beliefs associated with HIV/AIDS about themselves [8]. Finally, enacted stigma refers to the extent to which individuals living with HIV consider that they have experienced discrimination or prejudice from others in the community [9].

HIV is highly stigmatisable due to various reasons, most being misperceptions. For instance, it is considered contagious, severe, and resulting from norm violating volitional behaviour such as commercial sex work, homosexuality, and promiscuity [10, 11]. Besides it being dehumanising, HIV stigma presents a significant impediment to the adoption of HIV preventive behaviours such as voluntary disclosure of HIV status, HIV testing, and treatment adherence [6, 12] [13], thus causing a major setback to efforts made in the prevention and treatment of HIV/AIDs [14, 15]. Furthermore, the fact that adolescence is marked with rapid physical and psychological changes, coupled with unfamiliar demands amidst an increasing level of independence [16], suggests that adolescents may experience severe consequences arising from HIV stigma [17]. Furthermore, studies have found that perceived HIV stigma makes adolescents hide their status that needs to be well guarded due to the fear of rejection, isolation, and stigmatisation from others [17, 18]. Therefore, adolescents adopt either partial disclosure or non-disclosure strategies to avoid negative social consequences [17]. All these negatively impact both health-seeking behaviour and health outcomes. Although the negative impacts of stigma have been widely documented, the literature on HIV stigma has been majorly skewed towards adults living with HIV ignoring the impacts of stigma on adolescents living with HIV [18].

Scales to measure HIV stigma among adults have been developed and validated in high-income [19, 20] and lower-middle-income settings [21]. Berger’s 40-item HIV stigma scale [HSS-40] [19] is widely used as it captures the three stigma mechanisms (anticipated, internalised, enacted) for individuals living with HIV, as suggested by Earnshaw and Chaudoir [6]. Berger’s 40-item HIV stigma scale was originally developed and used in the USA [19]. It is a reliable and valid instrument for assessing HIV stigma among infected adults [19]. Several versions of the (HSS-40) have been adapted and used with children in Sweden [22] and young adults with HIV in Thailand [23] and the USA [24]. However, there is a lack of valid and reliable stigma measures, especially in resource-limited settings [25, 26]. Further, to our knowledge, there is a dearth of instruments that have been developed and validated for use with children living with HIV under the age of 17 years in the Kenyan context.

Research has shown that some of the lived experiences, underlying mechanisms, and perceptions surrounding stigma are similar among adolescents, young adults, and adults living with HIV [17, 27]. This finding’s implication is that stigma assessment tools or scales developed for adults living with HIV may potentially be useful for adolescents. However, before using these measures widely, the psychometric properties of these scales must be adequately examined. Therefore, the 12-item HIV stigma scale (HSS-12) version of the Berger HIV stigma scale [20] was used in the present study. HSS-12 has comparable psychometric properties to the full-length scale, and its brevity facilitates the inclusion of HIV stigma assessments into extensive surveys [20].

Given these knowledge gaps, the purpose of this quantitative study was to evaluate the psychometric characteristics (validity and reliability) and measurement invariance of the short version of the Berger HIV stigma scale to determine its usefulness for a longitudinal study among perinatally HIV adolescents from a rural coastal setting in Kilifi Kenya.

## Methods

### Study setting

The study setting’s details, participants, and recruitment processes have been previously described in detail [28]. A cross-sectional study with perinatally HIV-infected adolescents aged 12–17 years was conducted between November 2017 and October 2018 at the Centre for Geographic Medicine Research-Coast at the Kenya Medical Research Institute (CGMR-C/KEMRI). All participants were residents of Kilifi County on the coast of Kenya. Approximately 1.4 million people were Kilifi County residents by 2016, most (61%) residing in the rural areas [29]. Kilifi County is classified as a medium HIV county with a prevalence of 4.5%, of whom 19% are young people aged 19–24 years [30].

### Participants

We have used baseline data for an ongoing longitudinal study, the Adolescent Health Outcomes Study (AHOS). Two hundred and one (201) perinatally HIV-infected adolescents were enrolled and subsequently interviewed. Study participants were adolescents aged between 12 and 17 years at the time of recruitment, with confirmed HIV-positive status. They needed to be fully aware of their HIV status and that of their biological mother and provided written parental or guardian consent and adolescents' assent. All eligible adolescent participants had to be accompanied by a caretaker during their appointment for data collection at the CGMRC-KEMRI.

### Measures

#### HIV stigma

We adopted the 12-item HIV stigma scale (HSS-12) version of the Berger HIV stigma scale to assess the perceived stigma felt by perinatally HIV-infected adolescents. This tool was selected because of its confirmed comparable psychometric properties (reliability and validity) to the full-length scale, albeit short and simple [20]. The questionnaire has twelve items (see Table 2) categorised under four dimensions of stigma: (1) *personalised stigma,* perceived stigmatising consequences of others knowledge of an individual’s HIV status; (2) *disclosure concerns,* fear of self-disclosure, and fear that those who know would tell others; (3) *concerns with public attitudes,* conceptions of people about a person with HIV*; and* (4) *negative self-image,* experiencing oneself as infected and not as good as others each comprising a subscale of the *instrument* [22]. The 12 items are statements that a person living with HIV can agree or disagree with on a Likert scale rated as 1 “strongly disagree,” 2 “disagree,” 3 “agree,” and 4 “strongly agree.” Possible scores per item range from 1 to 4 (3–12 for sub-scale), and a total score ranging between (12 and 48) is derived from the summation of item scores. Higher scores indicate a higher level of perceived HIV stigma.

### Instrument translation

The HSS-12 was forward translated into Swahili by research team members fluent in English and Swahili and then back-translated into English by an independent back translator not involved in the project. The back-translated version of the tool was checked for comparability with the original English questionnaire [31]. In addition, members of the research team had a harmonisation meeting to review the questionnaire to ensure its cultural relevance to the study sample.

### Data collection procedures

Study participants were recruited through sequential sampling from all eligible and consenting families attending HIV clinics at eight health facilities in Kilifi County. In addition, perinatally HIV-infected adolescents and their caregivers were recruited by a trained research assistant in liaison with health workers at participating HIV treatment facilities.

A trained research assistant administered the Swahili version of the HIV stigma scale (HSS-12) to each study participant (in person) in a quiet private study clinic, using an android tablet. In addition, demographic information such as age, sex, education level, orphanhood, and clinical characteristics such as HIV viral load concentration and HIV clinical staging data were also collected [32, 33]. The data were entered in REDCap electronic database hosted at the KEMRI Wellcome Trust Programme. REDCap (Research Electronic Data Capture) is a secure, web-based software platform designed to support data capture for research studies, providing (1) an intuitive interface for validated data capture; (2) audit trails for tracking data manipulation and export procedures; (3) automated export procedures for seamless data downloads to common statistical packages; and (4) procedures for data integration and interoperability with external sources.

### Statistical analysis

Statistical analysis was performed for three psychometric properties of internal consistency, factor structure, and measurement invariance. The internal consistency was analysed using Cronbach’s alpha (α), whereby the value of α was considered acceptable if ≥ 0.7 [34, 35]. The factor structure was analysed using confirmatory factor analysis (CFA) based on a four-factor structure of the HSS-12. CFA was tested using weighted least squares mean and variance (WLSMV) using Lavaan [36] package in R statistics [37]. The criteria for a model fit were assessed using the chi-square test (χ^2^), Tucker-Lewis Index (TLI), Comparative Fit Index (CFI), and root mean square error of approximation (RMSEA). The criteria for acceptable fit was insignificant χ^2^ tests, a root mean square error of approximation (RMSEA) of < 0.05, a TLI, and a CFI of ≥ 0.90 [38]. An Exploratory Factor Analysis (EFA) using the principal component analysis (PCA) factor extraction method with oblimin rotation was carried out when the first CFA did not fit well. The Kaiser–Meyer–Olkin measure of sampling adequacy (KMO) and Bartlett’s test of sphericity were used to investigate data adequacy for factor analysis. Factor extraction was based on Kaiser’s criterion of retaining factors with eigenvalues of > 1 and visual exploration of the scree plot for breaks or discontinuities in the graphical representation of the eigenvalues [39]. We analysed the measurement invariance using the four CFA models with robust WLSMV to account for the stigma indicators' categorical nature across age and sex. Specifically, we assessed the change in CFI and the chi-square difference between the more and least constrained models based on scaling correction factors [40]. Measurement invariance was assumed when a change in CFI was ≤ 0.01 and when the chi-square was non-significant between successively more restricted models [41]. Frequencies (percentages) and median (with interquartile range [IQR]) were used to describe the sample characteristics. The confirmatory and exploratory factor analyses were conducted using Lavaan, SemTools, and Psych packages in R software version 4.0.2 [37]. All other statistical analyses were conducted using Stata version 14.0 statistical software package [42]. For all analyses, *p* ≤ 0.05 was considered statistically significant for all tests of the hypothesis.

## Results

### Participants’ characteristics

Participants’ socio-demographic characteristics are presented in Table 1. Overall, 201 perinatally HIV-infected adolescents attending treatment and care clinics at health facilities in Kilifi County were recruited and interviewed between November 2017 and October 2018. Respondents had a median age of 13 years (IQR = 12–15) ranging from 12 to 17. The vast majority of the respondents were in early adolescence [12–14 years] (69.7%). Slightly more than half (52%) were females and orphaned (51.2%) (either partial [a child with only one parent alive] or total). Most study participants were in stage 2 of the WHO clinical staging (77.2%). Perceived HIV stigma score ranged from 12 to 48 with a median score of 15 (IQR: 12–20).

**Table 1:** Participant’s sociodemographic and clinical characteristics

Sample characteristics	Total sample	
	n	%
*Sociodemographic characteristics*	201	
Age—years (12–17), median (IQR)	13 (12–15)	
Sex		
Female	105	52.2
Male	96	47.8
Adolescence stage		
Early adolescence (12–14 years)	140	69.7
Mid-adolescence (15–17 years	61	30.3
Education (number of years in formal education)—mean (SD)	1.8(0.5)	
Not attending school	2	1.0
Special school	1	0.5
Lower primary school (pre-primary—class 5)	100	50.5
Upper primary school (class 6–8)	81	40.9
Secondary school	14	7.1
Perceived HIV-stigma score^b^—median (IQR)	15(12–20)	
Orphanhood		
Both parents alive	98	48.8
Only mother alive	37	18.4
Only father alive	29	14.4
Both parents died	37	18.4
*Clinical characteristics*		
HIV viral load concentration		
≤ 1000 copies/mL	108	56.8
> 1000 copies/mL	82	43.2
WHO clinical stage, OM = 5		
Stage 1	10	5.1
Stage 2	142	72.1
Stage 3	45	22.8

*OM* observation with missing value, *SD* standard deviation, *a* score range = 0–9, *b* score range = 12–48, *IQR* interquartile range

### Analyses of the HSS-12

A summary of the participant’s scores on the HSS-12 is shown in Table 2. The median score was 3 (IQR: 1–5) for the personalised stigma subscale, 6 (IQR: 4–7) for the disclosure concern subscale, 3 (IQR: 1–5) for the public attitude’s subscale, 3 (IQR: 2–5) for the negative self-image subscale and 15 (IQR: 12–20) for the HSS-12 stigma scale. Individual items had medians and IQR ranging from 1 to 2 and 0 to 3, respectively.

**Table 2:** Descriptive statistics for the short-form version (HSS-12) of the HIV Stigma Scale

Item	Median item score^a^ (IQR)	Corrected item correlation	Total subscale score^b^ [Median, (IQR)]	Reliability α	Validity construct		
					CFI	RMSEA	TLI
*Personalised stigma*			3 (1–5)	0.68 (95% CI; 0.58–0.77)			
Some people stop touching me soon they know/realise I am infected with HIV/AIDS	1 (0–2)	0.63					
People I care for stopped calling me after knowing I suffer from AIDs	1 (0–2)	0.67					
I have lost friends for telling/explaining that I have AIDS	1 (0–2)	0.62					
*Disclosure concerns*			6 (4–7)	0.44 (95% CI; 0.30–0.58)			
Telling someone that I have AIDS is dangerous*	1 (0–2)	0.95					
I do all I can to keep my AIDS (HIV) status secret	2 (1–3)	0.24					
I am very careful to that person I tell about my HIV status (I am cautious/very careful to (?of) the people I tell my HIV status)	2 (2–3)	0.17					
*Concerns about public attitudes*			3 (1–5)	0.65 (95% CI; 0.55–0.76)			
People who are suffering from AIDS are treated as if they are not like the other people	1 (0–2)	0.63					
People believe that a person infected with HIV is dirty	1 (0–2)	0.66					
Many people are worried when they are near a person infected with HIV	1 (0–2)	0.60					
*Negative self image*			3 (2–5)	0.70 (95% CI; 0.61–0.79)			
I feel guilty because I am infected with HIV	1 (0–2)	0.64					
People’s attitudes about HIV/AIDS makes me feel very bad	1 (1–2)	0.66					
I feel I am not as good as others because am infected with HIV	1 (0–2)	0.68					
Overall			15 (12–20)	0.83 (95% CI; 0.79–0.87)	0.949	0.051	0.933

*IQR* interquartile range

^a^Possible score for each item 1–4; higher scores reflect a higher level of perceived HIV stigma

^b^Possible score 3–12 on each scale; higher scores reflect a higher level of perceived HIV-related stigma

#### Internal consistency and factor structure

The HSS-12 had an internal consistency reliability coefficient alpha = 0.83 (95% CI 0.79–0.87) (see Table 2). Corrected item-total correlation coefficients, an indicator of internal construct validity, had a range between 0.17 and 0.95, indicating that the broadness of the intended stigma concept had been captured. Despite the very good internal consistency for the full scale, the reliability of three subscales was low: personalised stigma α = 0.68 (95% CI; 0.58–0.77), disclosure concern α = 0.44 (95% CI; 0.30–0.58), and concerns with public attitudes α = 0.65 (95% CI; 0.55–0.76) sub-scales. Especially concerning was the extremely poor reliability of the disclosure concern subscale.

Confirmatory Factor Analyses of the HSS-12 showed a good fit with the original subscale structure. The χ^2^ test was statistically significant (χ^2^ = 75.804, *df* = 50, *p* = 0.011) and other model fit indices indicated that our data fit the four-factor model (RMSEA: 0.051; TLI: 0.933; CFI: 0.949). Although the model's goodness of fit was generally within the acceptable range, an EFA was conducted to abridge the scale and create a better model.

#### Exploratory factor analysis: creation of a new HSS model

**Fig. 1:**
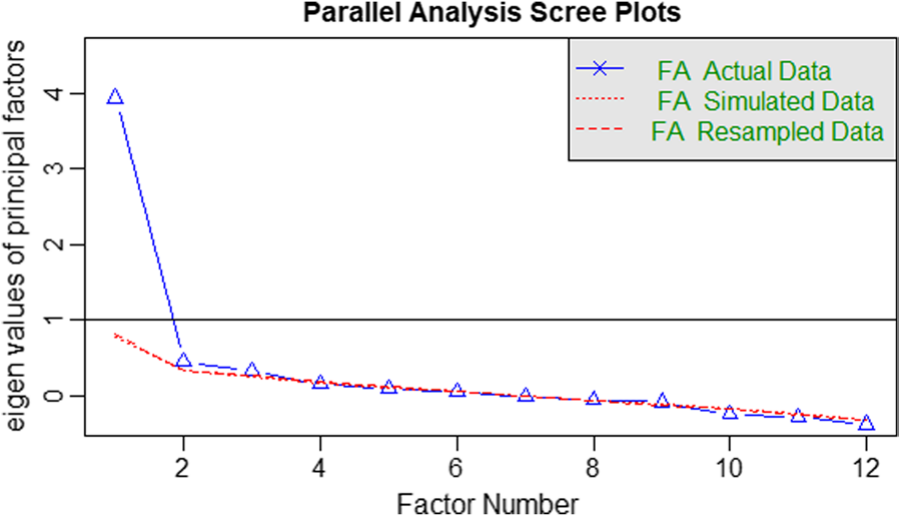
Scree plot showing eigenvalues from parallel analysis of the HSS-12

**Fig. 2:**
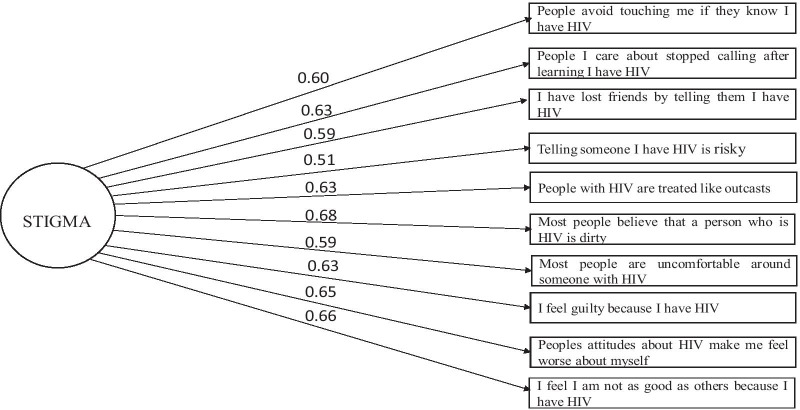
Confirmatory factor analysis of the unidimensional HSS-10. Sample (n = 195). Maximum likelihood estimates are standardised

We performed a parallel analysis (maximum likelihood) using a polychoric correlation matrix which suggested that the HSS-12 had only one factor with an eigenvalue > 1.0 (see Fig. 1), which accounted for 33.0% of the variance. Consequently, we conducted an exploratory factor analysis to clarify the HSS-12 structure. We examined factor loadings from the resultant EFA and dropped items with factor loadings of 0.4 or lower. Two items assessing “I do all I can to keep my AIDS (HIV) status secret” and “I am very careful to that person I tell about my HIV status” were dropped from the disclosure concern subscale due to low factor loadings. Factor analysis (oblimin rotation) revealed a unidimensional scale consisting of 10 items (see Fig. 2 for the factor loadings of the HSS-10 item abridged scale). Thus, the dropping of the two items improved scale reliability from 0.83 to 0.86.

### Analyses of the HSS-10 item abridged scale

#### Internal consistency

The HSS-10 had an internal consistency reliability coefficient alpha = 0.86 (95% CI 0.84–0.89) (see Table 3). The corrected item-total correlation coefficient ranged between 0.51 and 0.68, indicating that the intended stigma concept's broadness had been captured.

**Table 3:** Descriptive statistics for items in the abridged version (hss-10) of the hiv stigma scale

Item	Median item score^a^ (iqr)	Corrected item correlation	Total score [median (iqr)]	Reliability	Validity		
					Construct		
				α	CFI	RMSEA	TLI
Some people stop touching me soon they know/realise I am infected with HIV/AIDS	1 (0–2)	0.60					
People I care for stopped calling me after knowing I suffer from AIDs	1 (0–2)	0.63					
I have lost friends for telling/explaining that I have AIDS	1 (0–2)	0.59					
Telling someone that I have AIDS is dangerous*	1 (0–2)	0.51					
People who are suffering from AIDS are treated as if they are not like the other people	1 (0–2)	0.63					
People believe that a person infected with HIV is dirty	1 (0–2)	0.68					
Many people are worried when they are near a person infected with HIV	1 (0–2)	0.59					
I feel guilty because I am infected with HIV	1 (0–2)	0.63					
People’s attitudes about HIV/AIDS makes me feel very bad	1 (1–2)	0.65					
I feel I am not as good as others because am infected with HIV	1 (0–2)	0.66					
Overall			11 (7–16)	0.86 (95% CI; 0.84–0.89)	0.976	0.040	0.969

*IQR* interquartile Range

^a^Possible score for each item 1–4; higher scores reflect a higher level of perceived HIV stigma

#### Factor structure and measurement model by gender and age sub-groups

Confirmatory Factor Analysis tested the unidimensional HSS-10 model. The χ^2^ test was statistically insignificant (χ^2^ = 46.183, *df* = 35, *p* = 0.098). Additionally, other model fit indices indicated that our data fit the unidimensional model (RMSEA: 0.040; TLI: 0.969; CFI: 0.976).

Subsequently, four multi-Group Confirmatory Factor Analyses (MGCFA) were conducted separately for both sex and age sub-groups. All the models exhibited a good fit based on the CFI being greater than 0.90 (see Table 4). Furthermore, model fit based on RMSEA was best in the strict invariance model for both sex [RMSEA: 0.013 (90% CI 0.000–0.055)] and age [RMSEA: 0.037 (90% CI 0.000–0.067)], suggesting that constraining factor loadings, intercepts and variances improved model fit in the strict factorial invariance model compared to the configural, metric and scalar invariance models (see Table 4 for the details of the invariance results).

**Table 4:** Multi-group confirmatory factor analysis for age and gender sub-groups

Group	Invariance	χ^2^ (*df*)	*p* value	CFI^b^	TLI^b^	RMSEA^b^	Δχ^2^ (Δ*df*)	*p* value^a^	ΔCFI	ΔRMSEA
Age	Configural	80.86 (70)	0.176	0.974	0.967	0.040 [0.000–0.074]	–	–	0.007	–
	Metric/weak	92.89 (79)	0.136	0.967	0.962	0.042 [0.000–0.074]	10.98 (9)	0.2774	0.001	0.002
	Scalar/strong	101.34 (88)	0.157	0.968	0.967	0.039 [0.000–0.070]	8.77 (9)	0.4583	0.001	0.003
	Strict	111.06 (98)	0.173	0.969	0.971	0.037 [0.000–0.067]	10.60 (10)	0.3897	0.001	0.002
Sex	Configural	91.84 (88)	0.369	0.992	0.992	0.021 [0.000–0.060]	–	–	0.001	–
	Metric/weak	82.13 (79)	0.383	0.993	0.992	0.020 [0.000–0.061]	10.06 (9)	0.3452	0.001	0.001
	Scalar/strong	91.76 (88)	0.371	0.992	0.992	0.021 [0.000–0.060]	9.79 (9)	0.3681	0.001	0.001
	Strict	99.60 (98)	0.436	0.997	0.997	0.013 [0.000–0.055]	8.20 (10)	0.6094	0.005	0.008

^a^The chi-square difference value is not significant. It indicated that constraining the parameters of the nested model did not significantly worsen the fit of the model. Our result indicated measurement invariance

^b^Criteria for an acceptable fit were a root mean square error of approximation of < 0.06, and a comparative fit index (CFI) and a Tucker-Lewis index (TLI) of ≥ 0.90. Configural invariance—no constraints; Full metric invariance—with all factor loadings constrained equal. Scalar invariance—with all intercepts constrained equal; Strict invariance—with all factor loadings and intercepts fixed; Measurement invariance is assumed when ΔCFI is ≤ 0.01

## Discussion

Our study aimed to examine the psychometric properties of a short version of the HSS-40 [18], translated into Swahili using baseline data from a longitudinal study among perinatally HIV-infected adolescents. We evaluated the HSS-12 [20] reliability and construct validity, which indicated insufficient reliability on three of the four subscales. Especially concerning was the extremely poor reliability of the disclosure concern subscale. Accordingly, we conducted an exploratory factor analysis to improve scale structure. Our results indicated the need to exclude two items and create an abridged version of the scale (HSS-10). The EFA supported the scale's construct validity and resulted in a unidimensional 10-item scale measuring the construct stigma.

### Reliability and construct validity of the Swahili HSS-10

Two items from the disclosure concerns subscale with factor loadings < 0.4 were dropped, consequently improving the scale reliability. The Swahili version of the HSS-10 demonstrated adequate internal consistency reliability suggesting that the ten items in the questionnaire reflect the latent construct of HIV stigma. However, the two items could have had poor loading for various reasons. Firstly, the translation may have been inadequate, thus raising ambiguity. However, a robust approach was used to develop these translations and back translations; the exact translation has shown adequate reliability among adults [43]. Secondly, potentially, the two items were not developmentally appropriate for adolescents. Therefore, we recommend future studies to investigate why the two items had low factor loadings when used among adolescents.

### Factor structure and measurement model

Berger’s HIV stigma scale (HSS-40) [19] measures four dimensions of stigma: *personalised stigma, disclosure concerns, concerns with public attitudes, and negative self-image.* The initial version of the present study's questionnaire contained twelve items (HSS-12) covering all the four domains. However, the poor psychometric properties of two items measuring disclosure concerns subscale led to a reduction of the initial 12–item scale into the final 10-item unidimensional HIV stigma scale. The scale’s unidimensional structure is supported by high alphas and the large ratio of the 1st/2nd eigenvalues. This unidimensional structure confirms that the HSS-10 assesses a single underlying factor (HIV stigma) among our study population. This finding corroborates what has been reported in other studies. For instance, despite four factors emerging after EFA in the USA, extraction of one higher-order factor provided evidence of a single overall construct [19].

Additionally, we found that the one-factor solution explained 39% of the variance. However, HIV stigma is a multi-dimensional construct [20, 21, 24] that differs across cultures [21]. Therefore, the difference in the scale’s structure might be due to how different populations and cultures conceptualise HIV stigma or that adolescents might not conceptualise stigma as adults do.

### Measurement invariance test

Our results support the presence of a strict invariance according to age and sex, allowing meaningful group comparisons among perinatally HIV-infected adolescents at the Kenyan Coast. Therefore, we can confidently compare means and conclude that any difference between the unidimensional HSS-10 across sex and age groups comes from a real difference in HIV stigma and not from the measure's group-specific properties. Although various studies have used the 40–item HIV stigma scale [19] and the 12-item HIV stigma scale [20, 44] to assess stigma and reported their psychometric properties, no study has been found to report the measurement invariance of the tool. Therefore, future research involving HIV stigma assessment tools should use robust psychometric analytical models involving measurement invariance.

### Relevance in public health

Although several stigma scales exist, Berger et al.’s [19] 40-item HIV stigma scale is the most commonly used around the world that covers all stigma mechanisms affecting people [6]. Additionally, it presents solid evidence of validity and reliability [19]. However, to be included in more extensive surveys, a shorter instrument is preferred [20]. Improved brevity means that this tool may have beneficial clinical implications if included in routine care. It is less labour intensive yet can screen for a problem that significantly impedes HIV care and treatment. Our results support the use of the Swahili version of the HSS-10 among the Kenyan adolescent population. The evidence suggests the possibility of using HSS-10 among adolescents in other Swahili-speaking countries. Additionally, further adaptations could be made to the HSS-12 to understand why the two items failed, conducting cognitive interviews with adolescents to fully understand what else could be measured to capture their stigma experiences fully.

### Strengths and limitations of this study

The study’s strength is that it focused on the adolescent sub-population, which is rarely an area of focus. Moreover, we used robust psychometric analytical models that involve measurement invariance, an important aspect of structural validity. However, several limitations of this study must be considered when interpreting the findings and should be addressed in future studies. First, our results are based on a sample of perinatally HIV-infected adolescents attending a specialised HIV clinic in a rural context and who have already undergone the entire disclosure process. This might limit the generalizability of these findings to adolescents from urban settings who either attend a private hospital or have not undergone the full disclosure process and who have acquired HIV behaviorally. Secondly, we did not investigate various aspects of scale reliability (e.g., test–retest reliability of the Swahili version of the HSS-10 to ascertain scale stability over time). Future studies should explore the test–retest and inter-rater reliability of the HSS-10 when used among adolescents to ascertain scale stability over time. However, it is unlikely that the absence of test–retest reliability and inter-rater reliability in the present study had any major issues given that proper translation procedures of the HSS-10 to Swahili were observed and cognitive interviews from tool adaptation revealed that participants well comprehended the items of the Swahili version of HSS-10. Third, we did not examine invariance based on certain socio-economic measures such as household income because the study population is very homogenous, so there may be little differentiation to make. Future studies should investigate the potential implication of such factors on measurement invariance since socio-economic factors may influence HIV stigma. Lastly, we did not test for discriminant validity as we only collected data for the HIV stigma scale. Future research should consider assessing discriminant validity.

## Conclusion

This study presents a first published assessment of the HSS-12 in the adolescent population from East Africa. Evidence presented supports a unidimensional model and measurement invariance of the HSS-10 allowing for reliable comparisons between sex and age groups. Besides, measurement invariance is unlikely to be affected by differences in time-lapse, response styles, socio-economic factors, and interpretations of indicators. Furthermore, based on its validity and reliability, the HSS-10 is recommended as a useful tool for measuring HIV stigma among perinatally HIV-infected adolescents. Adolescents from the Kenyan coast appear to be experiencing stigma related to disclosure concerns than in the domains of personalised stigma, negative self-image, and concerns with public attitudes. Further research is needed to determine whether the psychometric soundness of the HSS-10 reported here would hold among perinatally HIV-infected adolescents from other regions for both females and males of different age groups and socio-economic status. Lastly, as this is the first study using the HSS-10, validation of this measure is vital in evaluating interventions to scale down HIV stigma in addition to its practical implication for future stigma research.

## Data Availability

Study data are available from the corresponding author upon reasonable request.

## Abbreviations

KMO: Kaiser Meyer–Olkin
PCA: Principal component analysis
MGCFA: Multi-group confirmatory factor analysis
EFA: Exploratory factor analysis
CFA: Confirmatory factor analysis
CGMR-C/KEMRI: Centre for Geographic Medicine Research-Coast at the Kenya Medical Research Institute
AHOS: Adolescents Health Outcomes Study
HIV: Human Immunodeficiency Virus
HSS: HIV stigma scale
CFI: Comparative Fit Index
TLI: Tucker Lewis Index
RMSEA: Root mean square error of approximation
SRMR: Standardised Root Mean Residual
ART: Antiretroviral therapy
REDCap: Research Electronic Data Capture
WLSMV: Weighted least squares mean and variance
IQR: Interquartile range
WHO: World Health Organization
CI: Confidence interval
